# Supplementary material for: Nonlinear Machine Learning in Warfarin Dose Prediction: Insights from Contemporary Modelling Studies
Source: J Pers Med. 2022 Apr 29;12(5):717. doi: 10.3390/jpm12050717 (PMC9147332; doi:10.3390/jpm12050717)
Supplement: Supplementary file 1 [file jpm-12-00717-s001.zip › jpm-1658163-supplementary.pdf]

Table S1. Preferred Reporting Items for Systematic Reviews and Meta-Analyses: The PRISMA Statement

**Section and Topic**	**Item**	**Checklist item**	**Location where item is reported**
**TITLE**			
Title	1	Identify the report as a systematic review.	Title page
**ABSTRACT**			
Abstract	2	Provide a structured summary including, as applicable: TITLE (Title); BACKGROUND (Objectives); METHODS (Eligibility criteria, Information sources, Risk of bias, Synthesis of results); RESULTS (Included studies, Synthesis of results); DISCUSSION (Limitations of evidence, Interpretation); OTHER (Funding, Registration)	Abstract
**INTRODUCTION**			
Rationale	3	Describe the rationale for the review in the context of existing knowledge.	Introduction
Objectives	4	Provide an explicit statement of the objective(s) or question(s) the review addresses.	Introduction
**METHODS**			
Eligibility criteria	5	Specify the inclusion and exclusion criteria for the review and how studies were grouped for the syntheses.	Methods (Search strategy and selection criteria)
Information sources	6	Specify all databases, registers, websites, organisations, reference lists and other sources searched or consulted to identify studies. Specify the date when each source was last searched or consulted.	Methods (Search strategy and selection criteria)
Search strategy	7	Present the full search strategies for all databases, registers and websites, including any filters and limits used.	**Table S2**

Table S1. Continued

Section and Topic	Item #	Checklist item	Location where item is reported
Selection process	8	Specify the methods used to decide whether a study met the inclusion criteria of the review, including how many reviewers screened each record and each report retrieved, whether they worked independently, and if applicable, details of automation tools used in the process.	Methods (Study selection and extraction of data)
Data collection process	9	Specify the methods used to collect data from reports, including how many reviewers collected data from each report, whether they worked independently, any processes for obtaining or confirming data from study investigators, and if applicable, details of automation tools used in the process.	Methods (Study selection and extraction of data)
Data items	10a	List and define all outcomes for which data were sought. Specify whether all results that were compatible with each outcome domain in each study were sought (e.g., for all measures, time points, analyses), and if not, the methods used to decide which results to collect.	Methods (Study selection and extraction of data)
	10b	List and define all other variables for which data were sought (e.g., participant and intervention characteristics, funding sources). Describe any assumptions made about any missing or unclear information.	Methods (Study selection and extraction of data)
Study risk of bias assessment	11	Specify the methods used to assess risk of bias in the included studies, including details of the tool(s) used, how many reviewers assessed each study and whether they worked independently, and if applicable, details of automation tools used in the process.	Methods (Adherence to reporting standards and risk of bias)
Effect measures	12	Specify for each outcome the effect measure(s) (e.g., risk ratio, mean difference) used in the synthesis or presentation of results.	Methods (Data synthesis)
Synthesis methods	13a	Describe the processes used to decide which studies were eligible for each synthesis (e.g., tabulating the study intervention characteristics and comparing against the planned groups for each synthesis (item #5)).	Methods (Data synthesis)
	13b	Describe any methods required to prepare the data for presentation or synthesis, such as handling of missing summary statistics, or data conversions.	Methods (Data synthesis)

Table S1. Continued

Section and Topic	Item	Checklist item	Location where item is reported
Synthesis methods	13c	Describe any methods used to tabulate or visually display results of individual studies and syntheses.	Methods (Data synthesis)
	13d	Describe any methods used to synthesize results and provide a rationale for the choice(s). If meta-analysis was performed, describe the model(s), method(s) to identify the presence and extent of statistical heterogeneity, and software package(s) used.	Methods (Data synthesis)
	13e	Describe any methods used to explore possible causes of heterogeneity among study results (e.g., subgroup analysis, meta-regression).	Methods (Data synthesis)
	13f	Describe any sensitivity analyses conducted to assess robustness of the synthesized results.	Methods (Data synthesis)
Reporting bias assessment	14	Describe any methods used to assess risk of bias due to missing results in a synthesis (arising from reporting biases).	Methods (Data synthesis)
Certainty assessment	15	Describe any methods used to assess certainty (or confidence) in the body of evidence for an outcome.	Methods (Data synthesis)
**RESULTS**			
Study selection	16a	Describe the results of the search and selection process, from the number of records identified in the search to the number of studies included in the review, ideally using a flow diagram.	Figure 1
	16b	Cite studies that might appear to meet the inclusion criteria, but which were excluded, and explain why they were excluded.	Figure 1
Study characteristics	17	Cite each included study and present its characteristics.	Results (General characteristics), Table S4
Risk of bias in studies	18	Present assessments of risk of bias for each included study.	Results (Methods and risk of bias), Table 1,

Table S1. Continued

Section and Topic	Item	Checklist item	Location where item is reported
Results of individual studies	19	For all outcomes, present, for each study: (a) summary statistics for each group (where appropriate) and (b) an effect estimate and its precision (e.g. confidence/credible interval), ideally using structured tables or plots.	Table S4
Results of syntheses	20a	For each synthesis, briefly summarise the characteristics and risk of bias among contributing studies.	Figure 3
	20b	Present results of all statistical syntheses conducted. If meta-analysis was done, present for each the summary estimate and its precision (e.g., confidence/credible interval) and measures of statistical heterogeneity. If comparing groups, describe the direction of the effect.	Not done
	20c	Present results of all investigations of possible causes of heterogeneity among study results.	Not done
	20d	Present results of all sensitivity analyses conducted to assess the robustness of the synthesized results.	Not done
Reporting biases	21	Present assessments of risk of bias due to missing results (arising from reporting biases) for each synthesis assessed.	Not done
Certainty of evidence	22	Present assessments of certainty (or confidence) in the body of evidence for each outcome assessed.	Not done
**DISCUSSION**			
Discussion	23a	Provide a general interpretation of the results in the context of other evidence.	Discussion
	23b	Discuss any limitations of the evidence included in the review.	Discussion
	23c	Discuss any limitations of the review processes used.	Discussion

Table S1. Continued

Section and Topic	Item	Checklist item	Location where item is reported
Discussion	23d	Discuss implications of the results for practice, policy, and future research.	Discussion
**OTHER INFORMATION**			
Registration and protocol	24a	Provide registration information for the review, including register name and registration number, or state that the review was not registered.	Not done
	24b	Indicate where the review protocol can be accessed, or state that a protocol was not prepared.	Not done
	24c	Describe and explain any amendments to information provided at registration or in the protocol.	Not done
Support	25	Describe sources of financial or non-financial support for the review, and the role of the funders or sponsors in the review.	Sources of Funding
Competing interests	26	Declare any competing interests of review authors.	Conflicts of Interest
Availability of data, code and other materials	27	Report which of the following are publicly available and where they can be found: template data collection forms; data extracted from included studies; data used for all analyses; analytic code; any other materials used in the review.	Data Availability Statement

Table S2. Search strategy used in PubMed, Embase, and Cochrane

**Literature databases**	**Search items**	**Items found**
PubMed	Vitamin K antagonist[Title/Abstract] OR warfarin[Title/Abstract] OR marevan[Title/Abstract]) OR coumadin[Title/Abstract] OR coumadine[Title/Abstract] OR dicoumarol[Title/Abstract] OR dicoumarin[Title/Abstract] OR acenocoumarol[Title/Abstract]<br>**AND**<br>model[Title/Abstract] OR algorithm[Title/Abstract] OR prediction[Title/Abstract] OR equation[Title/Abstract] OR calculation[Title/Abstract]	2592
Embase	‘Vitamin K antagonist’:ti,ab,kw OR ‘warfarin’: ti,ab,kw OR ‘marevan’: ti,ab,kw OR ‘coumadin’: ti,ab,kw OR ‘coumadine’: ti,ab,kw OR ‘dicoumarol’: ti,ab,kw OR ‘dicoumarin’: ti,ab,kw OR ‘acenocoumarol’: ti,ab,kw<br>**AND**<br>‘model’: ti,ab,kw OR ‘algorithm’: ti,ab,kw OR ‘prediction’: ti,ab,kw OR ‘equation’: ti,ab,kw OR ‘calculation’: ti,ab,kw	3118
Cochrane	Vitamin K antagonist: ti,ab,kw OR warfarin: ti,ab,kw OR marevan: ti,ab,kw OR coumadin: ti,ab,kw OR coumadine: ti,ab,kw OR dicoumarol: ti,ab,kw OR dicoumarin: ti,ab,kw OR acenocoumarol: ti,ab,kw<br>**AND**<br>model: ti,ab,kw OR algorithm: ti,ab,kw OR prediction: ti,ab,kw OR equation: ti,ab,kw OR calculation: ti,ab,kw	242

Table S3. PROBAST altered or excluded items

PROBAST item		Alteration for this study
3.1	Was the outcome determined appropriately?	If the study provided an explicit definition about outcome, or do not provide a clear definition, but report that patients were stable (based on a specified INR range) over a definite period of time, this section was answered 'Y' or 'PY', otherwise it was answered 'N' or 'PN'.
3.2	Was a prespecified or standard outcome definition used?	Not assessed (there is no generally agreed standard warfarin dose definition, and the appropriate definition of outcome is clearly stated in 3.1)
4.1	Were there a reasonable number of participants with the outcome?	For model development studies, if EPV >=200, this section was answered 'Y' or 'PY', otherwise it was answered 'N' or 'PN'.
4.2	Were continuous and categorical predictors handled appropriately?	Not assessed as not relevant for non-linear machine learning algorithms.
4.6	Were complexities in the data (e.g., censoring, competing risks, sampling of control participants) accounted for appropriately?	Not assessed as not relevant for non-linear machine learning algorithms.
4.7	Were relevant model performance measures evaluated appropriately?	For this review, we considered the following precision accuracy measures (Mean absolute error (MAE), Mean squared error (MSE), Root mean square error (RMSE), Coefficient of determination (R^2), Percentage of patients with ideal dose) to be appropriate, otherwise it was answered 'N' or 'PN'.
4.9	Do predictors and their assigned weights in the final model correspond to the results from the reported multivariable analysis?	Not assessed as not relevant for machine learning algorithms.

Table S4. Risk of Bias table: based on authors' judgements about each risk of bias item

Studies, Risk of bias	Participants		Predictors			Outcome					Analysis					
	Were appropriate data sources used, e.g., cohort, RCT, or nested case–control study data?	Were all inclusions and exclusions of participants appropriate?	Were predictors defined and assessed in a similar way for all participants?	Were predictor assessments made without knowledge of outcome data?	Are all predictors available at the time the model is intended to be used?	Was the outcome determined appropriately?	Were predictors excluded from the outcome definition?	Was the outcome defined and determined in a similar way for all participants?	Was the outcome determined without knowledge of predictor information?	Was the time interval between predictor assessment and outcome determination?	Were there a reasonable number of participants with the outcome?	Were all enrolled participants included in the analysis?	Were participants with missing data handled appropriately?	Was selection of predictors based on univariable analysis avoided?	Were relevant model performance measures evaluated appropriately?	Were model overfitting and optimism in model performance accounted for?
Solomon 2004, High risk	+	+	+	-	-	+	-	+	-	+	-	+	-	-	-	-
	Low		High			High					High					
Cosgun 2011, High risk	+	+	+	+	+	-	+	+	+	+	-	+	-	-	-	+
	Low		Low			High					High					
Hu 2012, High risk	+	+	+	+	+	+	+	+	+	+	-	+	-	NI	+	+
	Low		Low			Low					High					

Grossi 2014, High risk	+	+	+	+	+	+	+	+	+	+	-	+	-	+	+	+
	Low		Low			Low					High					
Saleh 2014, High risk	+	+	+	+	+	-	+	+	+	+	+	+	-	+	+	+
	Low		Low			High					High					
Zhou 2014, High risk	+	-	+	+	+	+	+	+	+	+	NI	+	-	-	+	+
	High		Low			Low					High					
Li 2015, High risk	+	+	+	+	+	-	+	NI	+	+	+	+	-	+	+	
	Low		Low			High					High					
Liu 2015, High risk	+	+	+	+	+	-	+	+	+	+	NI	+	-	+	+	+
	Low		Low			High					High					
Alzubiedi 2016, High risk	+	NI	+	+	+	-	+	+	+	+	-	+	-	+	+	+
	Unclear		Low			High					High					
Pavani 2016, High risk	+	NI	+	+	+	+	+	+	+	+	-	+	-	NI	+	-
	Unclear		Low			Low					High					
Li 2018, High risk	+	-	+	+	+	+	+	+	+	+	+	+	-	+	+	+
	High		Low			Low					High					
Ma 2018, High risk	+	+	+	+	+	-	+	NI	+	+	+	+	-	+	+	+
	Low		Low			High					High					
	+	-	+	+	+	+	+	+	+	+	+	+	-	-	+	+

Tao 2018, High risk	High		Low			Low					High					
Li 2019, High risk	+	-	+	+	+	+	+	+	+	+	+	+	-	-	+	+
	High		Low			Low					High					
Tao 2019, High risk	+	+	+	+	+	+	+	+	+	+	-	+	-	NI	+	+
	Low		Low			Low					High					
Tao 2019, High risk	+	+	+	+	+	+	+	+	+	+	-	+	-	+	+	+
	Low		Low			Low					High					
Roche-Lima 2020, High risk	+	+	+	+	+	+	+	+	+	+	-	+	-	NI	+	+
	Low		Low			Low					High					
Asiimwe 2021, High risk	+	+	+	+	+	+	+	+	+	+	-	+	+	+	+	
	Low		Low			Low					High					
Gu 2021, High risk	+	-	+	+	+	+	+	+	+	+	+	+	-	-	+	+
	High		Low			Low					High					
Liu 2021, High risk	+	-	+	+	+	-	+	+	+	+	-	+	+	-	+	+
	High		Low			High					High					
Ma 2021, High risk	+	-	+	+	+	+	+	+	+	+	+	+	-	-	+	+
	High		Low			Low					High					

Nguyen 2021, High risk	+	+	+	+	+	+	+	+	+	+	-	+	-	+	+	+
	Low		Low			Low					High					
Steiner 2021, High risk	+	-	+	+	+	+	+	+	+	+	NI	+	+	+	+	+
	High		Low			Low					Unclear					

NI, no information.

Table S5. Comparison of Non-linear machine learning algorithms and Linear regression algorithms in the same population for Warfarin dose prediction

Paper	NO. Patients	NO. Features	Variables	NO. Models		Predictive accuracy (mg/w)		Fit accuracy	
				LR	Non-linear ML	LR	Non-linear ML	LR	Non-linear ML
**Development**									
Solomon 2004	148	3	clinical predictors	1	1	NR	NR	r: 0.80	r: 0.82
Hu 2012	587	7	clinical predictors	1	9	MAE: 1.59	MAE: (1.47, 1.55)	NR	NR
Zhou 2014	1093	11	clinical predictors	1	1	MAE: 0.32 [#]	MAE:0.08 [#]	NR	NR
Liu 2015	4797	9	clinical + genetic predictors	2	7	MAE: 9.28	MAE: (8.84, 9.82)	NR	NR
Alzubiedi 2016	163	7	clinical + genetic predictors	1	1	MAE: 11.1	MAE: 11.2	R2: 0.38	R2: 0.38
Ma 2018	5743	13	clinical + genetic predictors	2	8	MAE: (8.52, 8.53)	MAE: (8.31, 10.86)	NR	NR
Tao 2019	289	7	clinical + genetic predictors	2	6	MSE:0.32[*]	MSE:(0.12, 0.22)	NR [*]	R2: (0.12, 0.44)
Tao 2019	617	11	clinical + genetic predictors	3	8	MAE: (5.80,5.87) [&]	MAE: (4.73,5.36) [&]	R2: (0.2,0.5)	R2: 0.44 [&]
Liu 2021	377	11	clinical + genetic predictors	3	3	MAE:(5.54,5.82)<br>MSE:(8.77, 9.71)<br>RMSE:(7.83, 8.23)	MAE: (2.98, 4.54)<br>MSE:(3.15, 5.21)<br>RMSE: (4.67, 6.03)	R2: (0.30, 0.36)	R2:(0.61,0.76)

Nguyen 2021	650	16/17	clinical + genetic predictors	1	1	MAE: 4.06<br>RMSE: 5.74	MAE: 4.48<br>RMSE: 6.3	r:0.73	r:0.77
Steiner 2021	7030	13	clinical + genetic predictors	1	3	MAE:8.13	MAE: (8.11, 8.18)	NR	NR
**Development with external validation (same data)**									
Li 2019 IV	13639	10	clinical predictors	1	1	MAE:4.88<br>MSE:4.03<br>RMSE:5.31	MAE:4.82<br>MSE:4.06<br>RMSE:5.33	NR	NR
Li 2019 EV	13639	10	clinical predictors	1	1	MAE:5.25<br>MSE:4.21<br>RMSE:5.43	MAE:5.18<br>MSE:4.20<br>RMSE:5.42	NR	NR
**Development with external validation (another data)**									
Asiimwe 2021	270	7	clinical predictors	8	13	MAE:(12.19, 13.11)	MAE:(12.07, 17.59)	NR	NR
**External validation**									
Li 2015	1295	10	clinical + genetic predictors	1	6	MAE: 4.39	MAE: (4.41, 4.76)	NR	NR
Li 2015	216	10	clinical + genetic predictors	1	6	MAE: 4.25	MAE: (4.40, 4.84)	NR	NR

Abbreviations: LR, linear regression algorithm; ML, machine learning algorithm; IV, internal validation group, EV, external validation group. # The value provided in literature was not clear, and it was impossible to distinguish whether it was derived from the training set or the test set. * There were two linear models (Yu model2, IWPC) in the included study, but only the data of Yu model2 was provided. & There were three linear models and eight Non-linear machine learning models in the included study, but only part of the data was provided.
